# Supplementary material for: Isolation and Identification of a Novel Phlebovirus, Hedi Virus, from Sandflies Collected in China
Source: Viruses. 2021 Apr 27;13(5):772. doi: 10.3390/v13050772 (PMC8145316; doi:10.3390/v13050772)
Supplement: Supplementary file 1 [file viruses-13-00772-s001.zip › Supplementary Materials 1.pdf]

Table S1. Primers used for whole-genome sequence amplification of HEDV1867-2.

Primer Name	Orientation	Sequence (5'-3')	Position*
L_F1	Sense	GACCATCTAGCGACCTCCACACACAAAG	1-8
L_R1	Antisense	ACTCAGGTTGTCGAAGCCAT	350-331
L_F2	Sense	GACAGGTCAACTGTTGGGTCA	180-200
L_R2	Antisense	CCTCTGTGACGTCCCAATCC	732-713
L_F3	Sense	GGATTGGGACGTCACAGAGG	713-732
L_R3	Antisense	TGGTCTGTCTAATGCGCCTG	1202-1183
L_F4	Sense	GTACGATGATCCTGAGGCCG	1145-1162
L_R4	Antisense	CTGTGGTCGCCTTATCCTCC	2004-1985
L_F5	Sense	AATGATGCACCACACCCTGA	1926-1945
L_R5	Antisense	TGTGCATTCCTCCTCTCAGC	2751-2732
L_F6	Sense	GGATGCCGTGTCTCATGCTA	2450-2469
L_R6	Antisense	TGCATCATCCCTGTGCTTGT	3268-3249
L_F7	Sense	AGACAACCACAGGGAGCTTG	3137-3156
L_R7	Antisense	CAGGCTCAATGTCACCCACT	4416-4397
L_F8	Sense	AGCCCCAAAGACAGGAGAAGA	4166-4186
L_R8	Antisense	GGTCTTGGTTTTCCCATCACTC	5054-5033
L_F9	Sense	ACCCAGCCATCACACTTGAG	4693-4712
L_R9	Antisense	CAACTCATCCTGCGGCATTG	5657-5638
L_F10	Sense	TAGGTCATGGGCTGAGGACA	5333-5352
L_R10	Antisense	TCACAAAGACCGCCCAATGA	6411-6393
L_F11	Sense	TCTTTGGGCCAGCTCATTTCA	6037-6057
L_R11	Antisense	GACCATCT AGCG ACCT CCAC ACACAAAG	6412-6405
M_F1	Sense	GACCATCT AGCG ACCT CCAC ACACAAAG	1-8
M_R1	Antisense	TCAGTGCAGTCAAAGACCGT	314-295
M_F2	Sense	AGGCGGCGTACATTGAAGAA	7-26
M_R2	Antisense	AACTCCTTTATGGTGCGCGA	728-709
M_F3	Sense	CAAGGTAGATAGCTCGCGCA	696-715
M_R3	Antisense	AGCAACATTTCAAACCCCGC	1163-1150
M_F4	Sense	CTGCGCCTAGACGAATGCTA	983-1002
M_R4	Antisense	CCATGGCTACATGCAACAGC	2105-2086
M_F5	Sense	GCCTGCAGGGTATCATGGTT	1506-1525
M_R5	Antisense	AAGCTTGGCACCTATTGCCT	2965-2946
M_F6	Sense	TTGAGCTGTAGGGAGGGTCA	2854-2873
M_R6	Antisense	GGGAGCCCTTAAGGTTGTGT	3120-3101
M_F7	Sense	TTTGAGCAGTGCGGAGGAAT	3037-3056
M_R7	Antisense	CTGTGCACCCAGGGTTTAGT	4251-4232

M_F8	Sense	GCGGACGAGAGGACTGTTTT	3754-3773
M_R8	Antisense	GACCATCT AGCG ACCT CCAC ACACAAAG	4408-4401
S_F1	Sense	GACCATCT AGCG ACCT CCAC ACACAAAG	1-8
S_R1	Antisense	TTGGAAACATGAGGCAAGGC	423-404
S_F2	Sense	CTCACTAGGGTGGCTTCTGC	362-381
S_R2	Antisense	GGCCGAATGGAGTTCCAGAT	910-891
S_F3	Sense	GCTTGCCACTTTGGGCATAA	703-722
S_R3	Antisense	TTATCGCTCCTGAGGGTGGT	1696-1677
S_F4	Sense	AAATGTTGTTGTGCCCCACC	1494-1513
S_R4	Antisense	GACCATCT AGCG ACCT CCAC ACACAAAG	1171-1164

* The positions of the primers corresponding to the nucleic acid sequence of the HEDV1867-2 genome.

Table S2. Strains information used in phylogenetic analyses.

	Species	Viruses	Strains	Date	Country	Host	GenBank Accession Numbers			
							RdRp	Polyprotein	nucleocapsid protein	nonstructural protein (NSs)
1	*Adana phlebovirus*	Adana virus	195	2012	Turkey	Phlebotomus sp.	YP_009227127	YP_009227128	YP_009227130	YP_009227129
2	*Aguacate phlebovirus*	Aguacate virus	VP-175A	\	Panama	Lutzomyia sp.	YP_004414703	YP_004414702	YP_004414705	YP_004414704
3	*Alcube phlebovirus*	Alcube virus	S20	2007	Portugal	Phlebotomus perniciosus	ALG75832	ALG75833	ALG75834	ALG75835
4	*Alenquer phlebovirus*	Alenquer virus	\	1976	Brazil	Homo sapiens	AEA30054	AEA30042	AEA30067	AEA30066
5	*Ambe phlebovirus*	Ambe virus	BeAr407981	1982	Brazil	Phlebotominae sp.	YP_009346030	YP_009346018	YP_009346032	YP_009346031
6	*Anhanga phlebovirus*	Anhanga virus	BeAn46852	1962	Brazil	Choloepus didactylus	YP_009346019	YP_009346033	YP_009346021	YP_009346020
7	*Arumowot phlebovirus*	Arumowot virus	\	\	Sudan	Culex antennatus	YP_009010958	YP_009010955	YP_009010957	YP_009010956
8	*Buenaventura phlebovirus*	Buenaventura virus	CoAr3319	1964	Colombia	sandfly	AKF42410	AKF42395	AKF42365	AKF42366
9	*Bujaru phlebovirus*	Bujaru virus	BeAn47693	1962	Brazil	Proechimys guyannensis	API68880	API68881	API68883	API68882
10	*Cacao phlebovirus*	Cacao virus	VP-437R	1970	Panama	Nyssomyia trapidoi	QCI62731	QCI62732	QCI62733	QCI62734
11	*Campana phlebovirus*	Campana virus	VP-334K	1970	Panama	sandfly	AKF42423	AKF42408	AKF42392	AKF42391

12	*Candiru phlebovirus*	Ariquemes virus	\	1988	Brazil	sandfly	AEA30056	AEA30044	AEA30069	AEA30068
		Candirú virus	\	1967	Brazil	Homo sapiens	YP_004347993	YP_004347992	YP_004347994	YP_004347995
		Jacundá virus	\	1984	Brazil	rodent	AEA29965	AEA29966	AEA29967	AEA29968
		Morumbi virus	\	1981	Brazil	Homo sapiens	AEA30061	AEA30049	AEA30081	AEA30080
		Mucura virus	\	1981	Brazil	mosquito	AEA30060	AEA30048	AEA30079	AEA30078
		Serra Norte virus	\	1991	Brazil	Homo sapiens	AEA30063	AEA30051	AEA30085	AEA30084
13	*Chagres phlebovirus*	Chagres virus	\	\	Panama	Homo sapiens	AEL29642	AEL29641	AEL29643	AEL29644
14	*Cocle phlebovirus*	Coclé virus	GML244915	2009	Panama	Homo sapiens	AKF42422	AKF42407	AKF42389	AKF42390
15	*Dashli phlebovirus*	Dāshlī virus	\	2011	Iran	Sergentomyia sp.	ALS88184	ALS88185	ALS88187	ALS88186
16	*Durania phlebovirus*	Durania virus	Co Ar 171162	1986	Colombia	Lutzomyia sp.	AEB70976	AEB70979	AEB70977	AEB70978
17	*Echarate phlebovirus*	Echarate virus	OBS 6528	1998	Peru	Homo sapiens	AEA30058	AEA30046	AEA30073	AEA30072
18	*Gabek phlebovirus*	Gabek Forest virus	Sud AN 754-61	1961	Sudan	Acomys cahirinus	AHK60944	AHK60929	AHK60935	AHK60936
19	*Gordil phlebovirus*	Gordil virus	Dak ANBr 496d	1984	Central African Republic	Lemniscomys striatus	AHK60943	AHK60928	AHK60934	AHK60933
20	*Icoaraci phlebovirus*	Icoaraci virus	BeAn24262	1960	Brazil	rodent	QCI62745	QCI62746	QCI62747	QCI62748

21	*Itaituba phlebovirus*	Itaituba virus	\	1971	Brazil	opossum	AEA30059	AEA30047	AEA30077	AEA30076
22	*Itaporanga phlebovirus*	Itaporanga virus	\	1962	Brazil	sentinel mouse	QCI62749	QCI62750	QCI62751	QCI62752
23	*Ixcanal phlebovirus*	Ixcanal virus	CA Ar 170897	\	Guatemala	Lutzomyia sp.	AEB70982	AEB70983	AEB70980	AEB70981
24	*Karimabad phlebovirus*	Karimabad virus	I-58	1959	Iran	Phlebotomus sp.	AHK60947	AHK60932	AHK60941	AHK60942
25	*La Gloria phlebovirus*	La Gloria virus	SP0584-PA-2014	2014	Panama	Psychodopygus panamensis	QBQ01748	QBQ01749	QBQ01751	QBQ01750
26	*Lara phlebovirus*	Rio Claro virus	Phlebovirus GGP-2011a	\	Venezuela	sentinel hamster	AEL29685	AEL29686	AEL29688	AEL29687
27	*Leticia phlebovirus*	Leticia virus	CoAr 171616	1987	Colombia	Sandfly	AEL29649	AEL29652	AEL29650	AEL29651
28	*Maldonado phlebovirus*	Maldonado virus	FMD 0077	2004	Peru	Homo sapiens	AEA30055	AEA30043	AEA30075	AEA30074.
29	*Massilia phlebovirus*	Massilia virus	127	2008	Portugal	Phlebotomus perniciosus	ALP32227	ALP32229	ALP32231	ALP32232
30	*Medjerda phlebovirus*	Medjerda Valley virus	T131	2010	Tunisia	Phlebotomus (Larroussius) sp.	ALX81176	ALX81177	ALX81179	ALX81178
31	*Mona Grita phlebovirus*	Mona Grita virus	SP0260-PA-2014	2014	Panama	Nyssomyia trapidoi	QBQ01754	QBQ01755	QBQ01757	QBQ01756
32	*Mukawa phlebovirus*	Mukawa virus	MKW73	2013	Japan	Ixodes persulcatus	BBE15818	BBE15815	BBE15816	BBE15817
33	*Munguba*	Munguba virus	\	1977	Brazil	*Nyssomyia*	YP_009346010	YP_009346034	YP_009346011	YP_009346012

	phlebovirus					*umbratilis*			
34	*Naples phlebovirus*	Arrábida virus	PoSFPhlebV/126/2008	2008	Portugal	Phlebotomine sandflies	YP_009224693	YP_009480530	YP_009480532	YP_009480531
		Granada virus	GR25	2004	Spain	sandfly	ADO17679*	ADO17680	ADO17682	ADO17681
		sandfly fever Naples virus	Toscana	\	\	\	YP_089669	YP_089671	YP_089668	YP_089667
35	*Nique phlebovirus*	Nique virus		1972	Panama	sandfly	AEA30062	AEA30050	AEA30083	AEA30082
36	*Ntepes phlebovirus*	Ntepes virus	MRG54-KE-2014	2014	Kenya	Sergentomyia sp.	AYC35226	AYC35223	AYC35224	AYC35225
37	*Odrenisrou phlebovirus*	Odrénisrou virus	\	\	Cote d'Ivoire	*Culex albiventris*	AEL29670	AEL29669	AEL29671	AEL29672
38	*Oriximina phlebovirus*	Oriximiná virus	\	\	Brazil	*Lutzomyia* sp.	AEA30065	AEA30053	AEA30089	AEA30088
39	*Pena Blanca phlebovirus*	Pena Blanca virus	SP1683-PA-2014	2014	Panama	Psychodopygus panamensis	QBQ01759	QBQ01760	QBQ01762	QBQ01761
40	*Punique phlebovirus*	Punique virus	PI-B4-2008	2008	Tunisia	Phlebotomus sp.	AFH88995	AFH88996	AFH88997	AFH88998
41	*Punta Toro phlebovirus*	Capira virus	VP-366G	1970	Panama	sandfly	AKF42424	AKF42409*	AKF42394	AKF42393
		Punta Toro virus	Adames	1970	Panama	Homo sapiens	YP_009512941	YP_009512940	YP_009512942	YP_009512943
42	*Rift Valley fever phlebovirus**	Rift Valley fever virus	ZH-548	1977	Egypt	Homo sapiens	YP_003848704	YP_003848705	YP_003848707	YP_003848706
43	*Rio Grande phlebovirus*	Rio Grande virus	TBM3-204	1973	USA	Neotoma micropus	QCI62753	QCI62754	QKN22591	QKN22591

44	*Saint Floris phlebovirus*	Saint-Floris virus	Dak ANB 512	1971	Central African Republic	Tatera sp.	AFH88999	AFH89000	AFH89001	AFH89002
45	*Salanga phlebovirus*	Salanga virus	AnB 904a	1971	Central African Republic	Aethomys medicatus	AGZ62535	AGZ62536	AGZ62538	AGZ62537
46	*Salehabad phlebovirus*	Arbia virus	ISS PHL18	1980	Italy	Phlebotomus perniciosus	AGA82737	AGA82738	AGA82739	AGA82740*
		Salehabad virus	I-81	1959	Iran	Phlebotomus sp.	AGA82741	AGA82742	AGA82743	AGA82744*
47	*Salobo phlabovirus*	Salobo virus	\	\	Brazil	*Proechimys guyannensis*	AED98383	AED98380	AED98382	AED9838
48	*Sicilian phlebovirus*	Sandfly fever Turkey virus	Izmir 19	2008	Turkey	Homo sapiens	YP_004382743	YP_004382742	YP_004382744	YP_004382745
49	*Tapara phlebovirus*	Tapara virus	BeAr413570	1983	Brazil	Phlebotominae sp.	YP_009346035	YP_009346022	YP_009346024	YP_009346023
50	*Tehran phlebovirus*	Tehran virus	I-47	1976	Iran	Phlebotomus perfiliewi	AFH89011	AFH89012	AFH89013	AFH89014
51	*Tico phebovirus*	Tico virus	SP0157-PA-2013	2013	Panama	Lutzomyia sp.	QBQ01764	QBQ01765	QBQ01767	QBQ01766
52	*Toros phlebovirus*	Toros virus	213	\	Turkey	Phlebotomus sp.	YP_009246447	YP_009480527	YP_009480529	YP_009480528
53	*Toscana phlebovirus*	Toscana virus	H4906	2004	France	Homo sapiens	\	AMY16462	AMY16463	AMY16464
54	*Tres Almendras phlebovirus*	Tres Almendras virus	SP0412-PA-2013	2013	Panama	Psychodopygus panamensis	QBQ01768	QBQ01769	QBQ01771	QBQ01770
55	*Turuna phlebovirus*	Turuna virus	\	1978	Brazil	sandfly	AEA30064	AEA30052	AEA30087	AEA30086

56	*Uriurana phlebovirus*	Uriurana virus	BeAr479776	1985	Brazil	Phlebotominae sp.	YP_009346036	YP_009346025	YP_009346038	YP_009346037
57	*Urucuri phlebovirus*	Urucuri virus	BeAn100049	1966	Brazil	Proechimys guyannensis	YP_009346026	YP_009346027	YP_009346029	YP_009346028
58	*Viola phlebovirus*	Viola virus	BR/MT_PanAr2015	2015	Brazil	Lutzomyia sp.	AWH90184	AWH90183	\	\
59	*Zerdali phlebovirus*	Zerdali virus	37	\	Turkey	Phlebotomus sp.	YP_009246413	YP_009480524	YP_009480526	YP_009480525
