# Supplementary material for: Isolation and Identification of a Novel Phlebovirus, Hedi Virus, from Sandflies Collected in China
Source: Viruses. 2021 Apr 27;13(5):772. doi: 10.3390/v13050772 (PMC8145316; doi:10.3390/v13050772)
Supplement: Supplementary file 1 [file viruses-13-00772-s001.zip › Supplementary Materials 2.pdf]

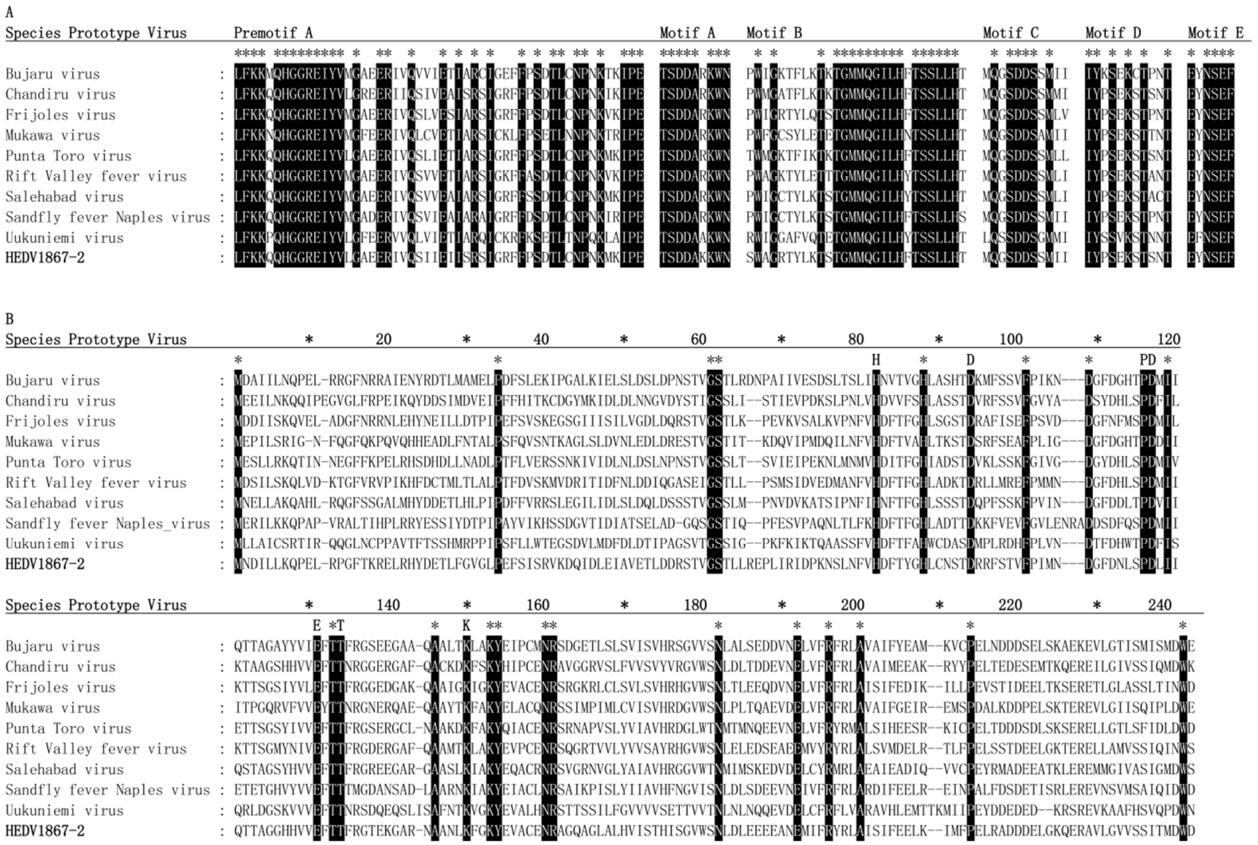

Figure S1. Sequence alignment of the conserved polymerase motifs (A) and endonuclease domain (B) of representative strains of *Phlebovirus* and HEDV1867-2. The conserved residues in the domain are identified by asterisks. The highly conserved endonuclease residues are shown in bold.

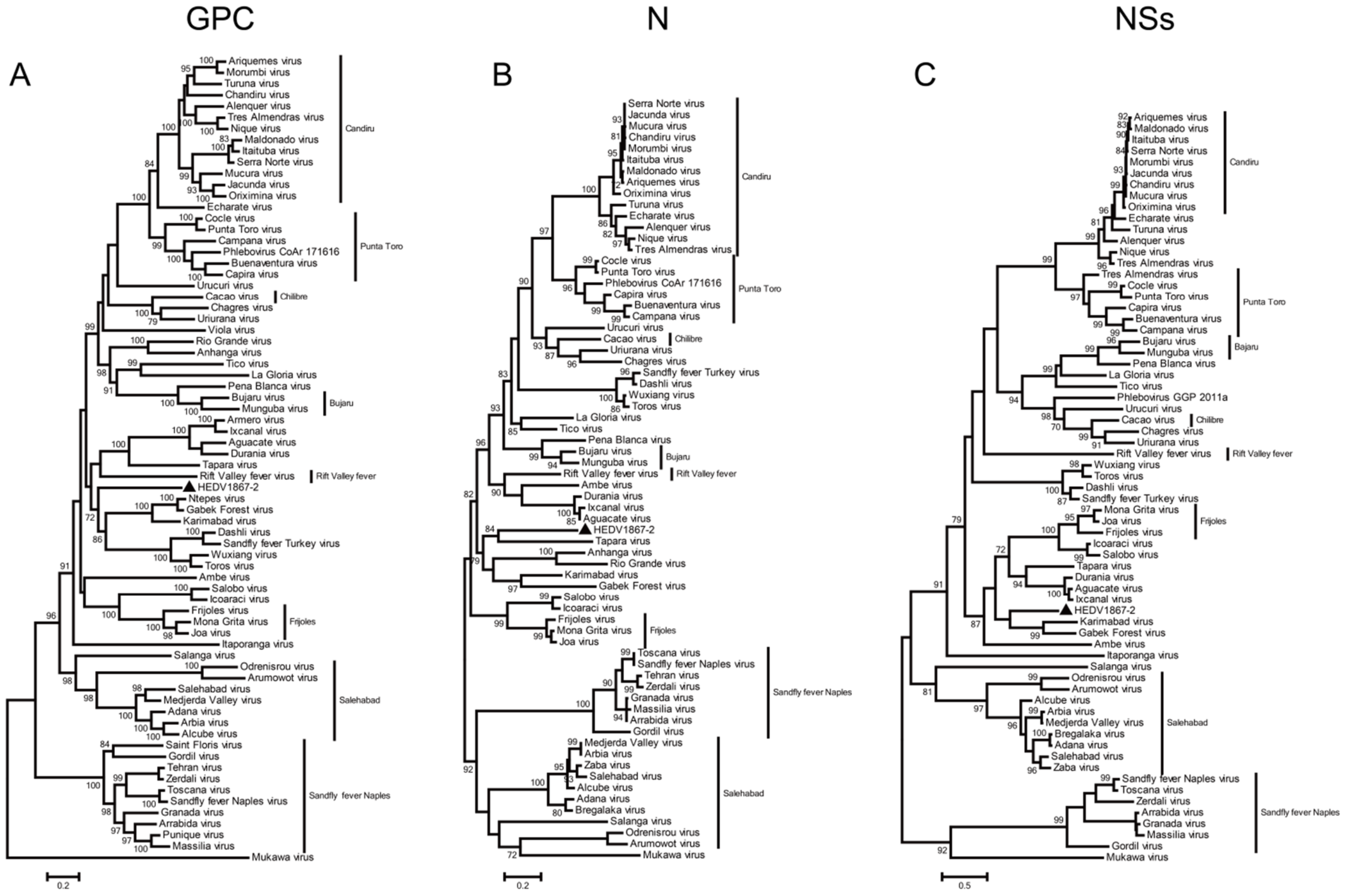

Figure S2. The evolutionary relationships of (A) GPC proteins, (B) N proteins, and (C) NSs proteins of HEDV1867-2 and 59 other phleboviruses based on maximum likelihood phylogenetic analysis. The HEDV1867-2 virus identified in this study was marked with black triangles.

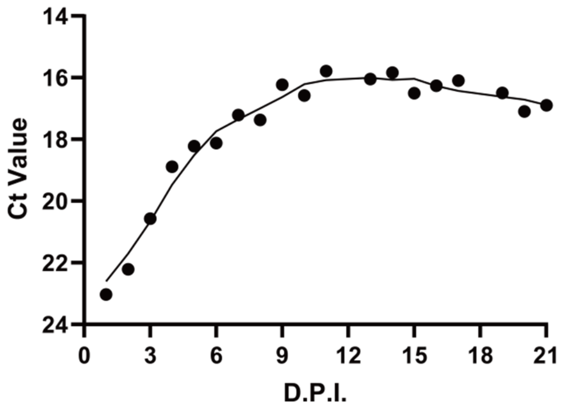

Figure S3. Proliferation curve of HEDV1867-2-infected BHK-21 cells.
